# Retraction: Myocardial Remodeling in Diabetic Cardiomyopathy Associated with Cardiac Mast Cell Activation

**DOI:** 10.1371/journal.pone.0305584

**Published:** 2024-06-11

**Authors:** The PLOS ONE Editors

After this article [1] was published, concerns were raised about Fig 4.

Specifically:

- There appear to be multiple regions of similarity in the backgrounds of all panels in Fig 4D.
- There appears to be a vertical discontinuity between lanes 3 and 4 in the MMP-9 panel in Fig 4D.

The authors did not respond to queries about these concerns. In the absence of underlying data, the above issues remain unresolved.

In light of the above unresolved concerns, which call into question the validity of these data, the *PLOS ONE* Editors retract this article.

All authors either did not respond directly or could not be reached.
